# Brazilian version of the Self-Estimated Functional Inability because of Pain questionnaire for musculoskeletal injuries relating to dance and sport: translation and cross-cultural adaptation

**DOI:** 10.1590/1516-3180.2019.0375.R1.08102019

**Published:** 2020-04-22

**Authors:** Jodimar Ribeiro dos, Jhonata Botelho Protázio, Aila Maria Muribeca-de-Castro, Jocassia Silva Pinheiro, Henrique Yuji Takahasi, Flavio de Oliveira Pires, Sergio Augusto Rosa de Souza, Cid André Fidelis-de-Paula-Gomes, Adriana Sousa Rêgo, Daniela Bassi-Dibai, Almir Vieira Dibai-Filho

**Affiliations:** I Undergraduate Student, Department of Physical Education, Universidade Federal do Maranhão (UFMA), São Luís (MA), Brazil.; II Undergraduate Student, Department of Physical Education, Universidade Federal do Maranhão (UFMA), São Luís (MA), Brazil.; III Undergraduate Student, Department of Physical Education, Universidade Federal do Maranhão (UFMA), São Luís (MA), Brazil.; IV Undergraduate Student, Department of Physical Education, Universidade Federal do Maranhão (UFMA), São Luís (MA), Brazil.; V PT. Master’s Student, Postgraduate Program on Physical Education, Universidade Federal do Maranhão (UFMA), São Luís (MA), Brazil.; VI PT. Professor, Department of Physical Education, Universidade Federal do Maranhão (UFMA), São Luís (MA), Brazil.; VII PhD. Professor, Department of Physical Education, Universidade Federal do Maranhão (UFMA), São Luís (MA), Brazil.; VIII PhD. Professor, Postgraduate Program on Rehabilitation Sciences, Universidade Nove de Julho, São Paulo (SP), Brazil.; IX PhD. Professor, Postgraduate Program on Program Management and Healthcare Services, Universidade Ceuma, São Luís (MA), Brazil.; X PhD. Professor, Postgraduate Program on Program Management and Healthcare Services, Universidade Ceuma, São Luís (MA), Brazil.; XI PhD. Professor, Postgraduate Program on Physical Education, Universidade Federal do Maranhão (UFMA), São Luís (MA), Brazil.

**Keywords:** Pain measurement, Reproducibility of results, Surveys and questionnaires, Cross-cultural adaptation, Dance injury, Sports injury

## Abstract

**BACKGROUND:**

Self-Estimated Functional Inability because of Pain (SEFIP) is a questionnaire specifically designed to measure musculoskeletal pain or discomfort.

**OBJECTIVE:**

To perform translation and cross-cultural adaptation of the SEFIP for dancers (SEFIP-dance), for use in Brazilian Portuguese. In addition, as a secondary objective, we adapted the translated version of SEFIP-dance for use among athletes or exercise practitioners (SEFIP-sport).

**DESIGN AND SETTING:**

Questionnaire translation and cross-cultural adaptation study conducted at a public university.

**METHODS:**

The Brazilian version of the SEFIP-dance questionnaire was developed following the processes of translation (involving two translators with Brazilian Portuguese as their mother tongue and fluency in English), backtranslation (involving two translators with English as their mother tongue and fluency in Brazilian Portuguese), committee review and pre-testing. SEFIP-sport was developed following the processes of content and face validation.

**RESULTS:**

SEFIP-dance was applied to 30 dancers, of mean age 22.38 years (standard deviation [SD] = 3.41), among whom 14 were men (46.66%). The participants understood 100% of the SEFIP-dance items and alternatives. SEFIP-sport was applied to 30 athletes or physical exercise practitioners, of mean age 25.09 years (SD = 8.93), among whom 25 were men (86.33%). The participants understood 100% of the ­SEFIP-sport items and alternatives.

**CONCLUSION:**

The Brazilian Portuguese versions of SEFIP-dance, translated and cross-culturally adapted for dancers, and SEFIP-sport, adapted for athletes or physical exercise practitioners, were shown to have adequate levels of understanding.

## INTRODUCTION

Cross-cultural adaptations of questionnaires in developing countries, such as Brazil, have fostered a major debate involving the fields of economics, health, politics and culture.^1^ Today, with the development and dissemination of the Guidelines for the Process of Cross-Cultural Adaptation of Self-Report Measures^2^ and of the Consensus-based Standards for the Selection of Health Measurement Instruments (COSMIN),^3^ standardization of cross-cultural adaptation relating to culture, language and country is providing positive outcomes within scientific and clinical contexts.

Within healthcare sciences, especially in the field of prevention and rehabilitation of musculoskeletal injuries, it is common to use questionnaires to measure self-reported outcomes, mainly in relation to pain and functional disability.^4^^,^^5^^,^^6^ Among the questionnaires for screening of musculoskeletal injuries, in addition to instruments that were created by researchers for specific evaluations,^7^^,^^8^ the Nordic Musculoskeletal Questionnaire (NMQ) stands out through its widespread use for locating musculoskeletal pain in diverse populations.^9^^,^^10^^,^^11^^,^^12^ However, the NMQ does not have a severity score, and it is not possible to use it to make inferences about functional disability.

Therefore, as a way to fill this gap, the Self-Estimated Functional Inability because of Pain (SEFIP) questionnaire was developed and published in 1999. This is an instrument created based on the NMQ but with the addition of severity grades relating to functional disability.^13^ However, the SEFIP questionnaire was specifically designed to measure musculoskeletal pain or discomfort in dancers. It has nonetheless been used in important studies^14^^,^^15^ and has already been translated and validated for the Turkish language.^16^

However, there is no validated questionnaire in the Portuguese language to measure pain and screen for musculoskeletal injuries specifically in dancers. Thus, there is justification for such a study. In addition, since the SEFIP questionnaire has a broad and usable structure for individuals who are not involved in dance, we highlight the importance of performing adaptations for its use in other populations, such as athletes^14^ and exercise practitioners,^8^ who commonly present musculoskeletal pain.

Given the above, the primary objective of this study was to perform the translation and cross-cultural adaptation of the SEFIP questionnaire for dancers (SEFIP-dance), for use in Brazilian Portuguese. In addition, as a secondary objective, we adapted the translated version of SEFIP-dance for use among athletes or exercise practitioners (SEFIP-sport).

## METHODS

### Study design

This was a cross-sectional study on translation and cross-cultural adaptation of a questionnaire. It was conducted in accordance with the Guidelines for the Process of Cross-cultural Adaptation of Self-Report Measures^2^ and the COSMIN.^3^ After our institution’s research ethics committee had approved the procedures for the study (through opinion number 3.051.824; date: December 3, 2018), the study was conducted at this public university. The recruitment of participants took place in communities around the university by means of verbal disclosure, posters and social media. All participants included in the study validated their participation by signing a free and informed consent statement.

### Translation and cross-cultural adaptation of SEFIP-dance

The process of translation and cross-cultural adaptation of SEFIP-dance for Brazilian Portuguese followed the criteria of Beaton et al.^2^ and was performed in five stages, as described below.

1. Translation: Two independent translators, comprising one physiotherapist with 10 years of experience in rehabilitation (T1) and one English teacher with 21 years of experience in translation without technical knowledge of the field of healthcare (T2), with Brazilian Portuguese as their mother tongue and fluency in English, translated the original version of SEFIP-dance into Brazilian Portuguese.
2. Synthesis of translations: After discussions and revisions, the two translators, under observation by one of the researchers, synthesized the two independently translated versions of the questionnaire (T1 and T2) and produced a single consensual version of SEFIP-dance (T12).
3. Back-translation: Two independent translators (without technical knowledge of the field of healthcare), both with English as their mother tongue and fluency in Portuguese, translated the Portuguese version of SEFIP-dance back into English, without previous knowledge of the original version of the questionnaire (B1 and B2). These translators were not the same as those in phase 1 (English to Portuguese language translation).
4. Expert committee review: Four rehabilitation experts, together with the four translators involved in the project, reviewed all the translated and back-translated versions for corrections of possible discrepancies, thus reaching the pre-final version of the rehabilitation project. At this stage, the criteria for including rehabilitation experts were as follows: time availability, fluency in both languages, clinical expertise with dancers and interest in collaborating in the study. The pre-final version of SEFIP-dance was agreed among all the committee members.
5. Pre-final test: The pre-final version of SEFIP-dance was applied to 30 dancers with pain in any body region and with Brazilian Portuguese as their mother tongue. The participants read and completed the questionnaire and, at the end of the questionnaire, established that they had understood the pre-final version of SEFIP-dance by selecting check boxes containing “yes” or “no” answers to each question on the questionnaire. If questions were not understood by more than 20% of participants, it was established that they would be reworded and retested among new samples of 30 participants each,^17^ until the desired level of understanding was reached, thus arriving at the final version of SEFIP-dance in Brazilian Portuguese.

### Sample for adaptation of SEFIP-dance

We used the following eligibility criteria for selecting the adaptation sample: professional dancers and/or dancers who used dance as a recreational activity, with a weekly frequency of at least twice a week; with the ability to read and write in Brazilian Portuguese; without cognitive impairment; and aged 18 years or older.

### Adaptation for SEFIP-sport

Initially, two researchers who were directly involved in the SEFIP-dance translation and cross-cultural adaptation process made changes to SEFIP-dance to readdress the questions and items of the questionnaire for athletes and sports practitioners (SEFIP-sport). This first version of SEFIP-sport was then submitted for face and content validation in two phases.^18^

First phase, two physical therapists and two physical education professionals working in the field of sports rehabilitation were consulted with regard to making technical judgments about alterations, inclusions or exclusions of items in the questionnaire and establishing whether the questionnaire was adequate for measuring musculoskeletal pain-related disability. The criteria for including these experts were the following: time availability, fluency in both languages, clinical and scientific expertise with sports rehabilitation and interest in collaborating in the study.

Second phase, five healthcare professionals were consulted to provide information about possible difficulties in reading the questionnaire and regarding the level of understanding of the items, clarity of response alternatives, presence of typographical errors, size of the letters, length of the questionnaire, time taken for application and overall evaluation of the questionnaire. The criteria for including these experts were the following: time availability, fluency in both languages, clinical expertise with sports rehabilitation and interest in collaborating in the study.

After the face and content validity had been established, the pre-final version of SEFIP-sport was applied to 30 regular practitioners of any sport (who had been doing this for at least 6 months), aged 18 years or older, with Brazilian Portuguese as their mother tongue. The evaluation procedures for the pre-final version of SEFIP-sport followed the same principles as used for SEFIP-dance, thus reaching the final version of SEFIP-sport.

### Scoring for SEFIP-dance and SEFIP-sport

Each questionnaire consists of 14 items, each relating to one body part, and it is possible to mark one of five answers for each item, which correspond to scores from 0 to 4. Thus, the total score ranges between 0 and 56 points.^13^ The higher the score is, the greater the disability also is. However, to avoid errors in interpreting the magnitude of functional disability through the total score, we suggest that separate analysis should be conducted on each body part, thus resulting in scores ranging from 0 (no pain and disability) to 4 (maximum pain and disability).

### Statistical analysis

The data collected during the pre-final test phase were analyzed descriptively through presentation of quantitative variables by means of averages (with standard deviation [SD]) and categorical variables by means of absolute numbers (with percentages). Data processing was performed using the SPSS software, version 17.0 (Chicago, IL, USA).

## RESULTS

### Translation and cross-cultural adaptation of SEFIP-dance

The translation and back-translation processes are described in Table 1. From this process, the pre-final version of SEFIP-dance was defined by the committee of experts. This version was then applied to 30 dancers whose mother tongue was Brazilian Portuguese. The average age of the dancers was 22.38 years (SD = 3.41), and 14 of these participants were men (46.66%). The most common dance styles practiced were hip-hop (n = 10; 33.33%), jazz (n = 9; 30%), ballroom (n = 6; 20%) and ballet (n = 5; 16.67%).

**Table 1 .** Translation, consensus version and backtranslation of the Self-Estimated Functional Inability because of Pain (SEFIP) questionnaire for dancers

SEFIP original	Translation	Consensus version	Backtranslation
Item			
1. Neck	T1: Pescoço T2: Pescoço	T12: Pescoço	B1: Neck B2: Neck
2. Shoulders	T1: Ombros T2: Ombros	T12: Ombros	B1: Shoulders B2: Shoulders
3. Elbows	T1: Cotovelos T2: Cotovelos	T12: Cotovelos	B1: Elbows B2: Elbows
4. Wrists/hands	T1: Punhos/mãos T2: Punhos/mãos	T12: Punhos/mãos	B1: Wrists/hands B2: Wrists/hands
5. Upper back	T1: Parte superior das costas T2: Parte superior das costas	T12: Parte superior das costas	B1: Upper back B2: Upper back
6. Lower back	T1: Parte inferior das costas T2: Parte inferior das costas	T12: Parte inferior das costas	B1: Lower back B2: Lower back
7. Hips	T1: Quadris T2: Quadris	T12: Quadris	B1: Hips B2: Hips
8. Thighs (front)	T1: Coxas (frente) T2: Coxas (parte anterior)	T12: Coxas (frente)	B1: Thighs (front) B2: Thighs (front)
9. Thighs (back)	T1: Coxas (atrás) T2: Coxas (parte posterior)	T12: Coxas (atrás)	B1: Thighs (back) B2: Thighs (back)
10. Knees	T1: Joelhos T2: Joelhos	T12: Joelhos	B1: Knees B2: Knees
11. Shins	T1: Canelas T2: Pernas (parte anterior)	T12: Pernas (frente)	B1: Legs (front) B2: Legs (front)
12. Calves	T1: Panturrilhas T2: Panturrilhas	T12: Panturrilhas	B1: Calves B2: Calves
13. Ankles/feet	T1: Tornozelo/pés T2: Tornozelos/pés	T12: Tornozelos/pés	B1: Ankles/feet B2: Ankles/feet
14. Toes	T1: Dedos dos pés T2: Dedos (do pé)	T12: Dedos dos pés	B1: Toes B2: Toes
Score			
0. Very well.	T1: Sem dor. T2: Muito bem.	T12: Sem dor.	B1: Without pain. B2: No pain.
1. Some pain but not much problem.	T1: Alguma dor, mas sem muitos problemas. T2: Alguma dor, mas não tem muito problema.	T12: Alguma dor, mas sem muitos problemas.	B1: Some pain, but without many problems. B2: Some pain, but without many problems.
2. Pretty much pain but can handle it.	T1: Bastante dor, mas consigo aguentar. T2: Muita dor, mas eu consigo lidar com isso.	T12: Bastante dor, mas eu consigo suportar.	B1: Quite painful, but bearable. B2: Quite a bit of pain, but I can handle it.
3. Much pain, must avoid some movements.	T1: Muita dor, preciso evitar certos movimentos. T2: Muita dor e devo evitar alguns movimentos.	T12: Muita dor, eu evito certos movimentos.	B1: A lot of pain, I avoid certain movements. B2: A lot of pain, I avoid certain moves
4. Cannot work in the production because of pain.	T1: Não consigo dançar por causa da dor. T2: Não consigo trabalhar na produção por causa da dor.	T12: Não consigo dançar por causa da dor.	B1: I am not able to dance because of the pain. B2: I cannot dance because of the pain.

T1 = Translation 1; T2 = Translation 2; T12 = Consensual synthesis of translations 1 and 2; B1 = Backtranslation 1; B2 = Backtranslation 2.

The participants understood 100% of the SEFIP-dance items and alternatives, and no changes to the pre-final version were required. The mean total score for SEFIP-dance was 5.63 (SD = 4.66). The “lower back” item was the one most often marked (n = 20; 66.66%), with a mean score of 1.06 (SD = 0.94). The final version of the SEFIP-dance questionnaire in Brazilian Portuguese is presented in Appendix 1.

### Adaptation for SEFIP-sport

The adaptation for SEFIP-sport was performed based on the version of SEFIP-dance that had been translated and cross-culturally adapted for the Brazilian population. Initially, the following three changes were made to the questionnaire: the alternative with score 4 was changed from *“Não consigo dançar por causa da dor”* (“I cannot dance because of the pain”) to *“Não consigo praticar o esporte por causa da dor”* (“I cannot practice the sport because of the pain”); item 13 was changed from *“Tornozelos/pés”* (“Ankles/feet”) to *“Tornozelos”* (“Ankles”); and item 14 was changed from *“Dedos dos pés”* (“Toes”) to *“Pés”* (“Feet”). In dance, a specific item *“dedos dos pés”* (“toes”) is justified due to the common use of this body part, especially in styles such as ballet. However, for various sports, the term “feet” was considered broader and more accurate for SEFIP-sport. The version submitted for face and content validity was approved by 100% of the experts, with no disagreements or suggestions for changes in SEFIP-sport.

Thus, the version approved after the face and content validation was considered to be the pre-final version of SEFIP-sport. Thirty athletes or physical exercise practitioners whose mother tongue was Brazilian Portuguese answered the questionnaire. The mean age of the athletes or exercise practitioners was 25.09 years (standard deviation [SD] = 8.93), and 25 of these participants were male (86.33%). The most common sports practiced were jiu-jitsu (n = 9; 30%), futsal (n = 5; 16.68%), athletics (n = 4; 13.33%), basketball (n = 4; 13.33%), volleyball (n = 4; 13.33%) and karate (n = 4; 13.33%).

The participants understood 100% of the SEFIP-sport items and alternatives, and no changes to the pre-final version were required. The mean total score for SEFIP-sport was 5.07 (SD = 4.25). The item “knee” was the one most often marked (n = 15; 50%), with a mean score of 0.70 (SD = 0.48). The final version of the SEFIP-sport questionnaire in Brazilian Portuguese and English (free translation) are presented in Appendices 2 and 3.

## DISCUSSION

We performed the translation and cross-cultural adaptation of SEFIP-dance for use in Brazilian Portuguese as the first step in the validation process for this questionnaire. This will allow its future use for investigating musculoskeletal injuries in dancers. In addition, because of the broad characteristics of SEFIP-dance, we were able to change and adjust the translated and adapted version of SEFIP-dance for use among athletes and exercise practitioners (SEFIP-sport).

There are several specific questionnaires in the scientific literature that address complaints relating to certain parts of the body, such as the knee,^19^ hip,^20^ ankle and foot,^21^ shoulder,^22^ hand and wrist,^17^ cervical spine^23^^,^^24^ and lumbar spine.^4^ However, to assess the presence of musculoskeletal pain or discomfort throughout the body, the NMQ remains the tool most commonly used in research and clinical practice.^8^^,^^11^^,^^12^^,^^25^^,^^26^

The NMQ makes it possible to record which part of the subject’s body has experienced pain in the last 12 months and 7 days, thus generating a nominal result.^27^ In a complementary manner, SEFIP-dance and SEFIP-sport not only allow recording of which body region presented pain at a given moment but also have a disability scale ranging from 0 to 4 points, thereby generating a numerical score for interpretations of pain and disability.^13^ Furthermore, it is noteworthy that the two questionnaires adapted here (SEFIP-dance and SEFIP-sport) are easy to understand, since 100% of the sample included in the pre-final evaluation phase understood all the questions.

This study has limitations that need to be considered. SEFIP-dance was translated and cross-culturally adapted based on Beaton et al.^2^ and was adapted for practitioners of any sport, based on content and face validity,^18^ thus creating a new questionnaire called the SEFIP-sport. Performing only cross-cultural translation and adaptation is common in the scientific literature.^24^^,^^28^^,^^29^ However, these procedures are only the first phase in properly ascertaining the validity of these questionnaires. Therefore, we recommend that further studies should be conducted to verify the psychometric properties of SEFIP-dance in Brazilian Portuguese and SEFIP-sport in Brazilian Portuguese and English.

## CONCLUSION

The Brazilian Portuguese versions of SEFIP-dance, translated and cross-culturally adapted for dancers, and SEFIP-sport, adapted for athletes or physical exercise practitioners, were shown to have adequate levels of understanding in the target population for each questionnaire.
